# Questionnaire about psychology/disease correlation–I

**Published:** 2011-02-25

**Authors:** D Dragoş, DG Ojog, OM Pănescu, EC Rusu, MD Tănăsescu

**Affiliations:** * ‘Carol Davila’ University of Medicine and Pharmacy BucharestRomania; ** 1^st^ Internal Medicine Department, University Emergency Hospital BucharestRomania; *** ‘Psyho Global Consult’ Ltd., BucharestRomania; **** ‘Spiru Haret’ University, Faculty of Sociology–Psychology, BucharestRomania

**Keywords:** personality inventory, psychological profile, psychological predisposition to disease

## Abstract

**Rationale**: The existing personality inventories are exploring too general psychological features so that the possible psychology/disease associations might be leveled out.

**Objective**: We attempt to build a tool to explore the possible correlation between certain psychological features and the most common internal disorders.

**Method**: We have used two questionnaires containing many pairs of synonymous items (necessary for assessing the consistency of the answers). The items are divided into four main domains: preoccupation for the basal conditions of existence (health/ disease/ death, fear, money, lodging); interaction with other people; action, will/ volition, self-assertion; and preoccupation with the exterior. In this first article we are presenting the correlations between items of the first domain, based on the answers from our first 3138 respondents.

**Results and discussion**: The concern about health is best reflected by general formulations. The desire for security is best expressed by items combining the worry about money and dwelling, and worst by items reflecting the eagerness to gain, keep or judiciously spend money. Among the various fears, those of future, darkness, and loneliness are better indicators of security concern. In assessing the anxiety about safety/ security, specific worries are more revelatory than the general ones. Precaution and inclination for order are the best indicators for the aspiration to stability. Poorer ones are the desire for cleanliness and the tendency to attachment. Health and security concerns seem to be consistently linked. The consistency evaluating system will be based upon pairs of synonymous items correlated with a10^–200^ or less error probability

**Abbreviations**: PP = psychological profile; PF = personality feature; Q1/ Q2/ Q3 = first/ second/ third questionnaire; HeSD = health subdomain; SeSD = security subdomain; StSD = stability subdomain; ChiSq = chi square; ErrProb = error probability (probability of error).

## Introduction

Presently, there is a large amount of data concerning the involvement of psychic factors in the pathogenesis of medical disorders. Disconcerting is their lack of specificity. On one hand, a given category of diseases seems to be associated with a whole spectrum of psychic traits. For example, cardiovascular disorders are associated with despair/ despondency, pessimism, depression, rumination, anxiety, anger, hostility [1]. On the other hand, each of the main psychic disorders seems to be related to a great variety of illnesses. Depression seems to hold the record–it has been associated with a various functional and organic disorders from many different pathological areas, especially cardiovascular diseases/ events [2–12], but also respiratory (such as functional respiratory complaints [13] pulmonary hypertension [14], chronic bronchitis [15]), digestive (irritable bowel syndrome [16, 17], inflammatory bowel disease [18], chronic hepatitis C [19], nonalcoholic steatohepatitis [20]), endocrine (diabetes [21, 22], polycystic ovary syndrome [23], musculoskeletal (chronic musculoskeletal pain [24], fibromyalgia [25, 26], temporomandibular joint syndrome [27], inflammatory rheumatic diseases [28]), immunological (allergy [29], chronic urticaria [30]), general (chronic fatigue syndrome [31]).

On its turn, anxiety has been associated with respiratory conditions, such as asthma [32, 33, 34] and chronic bronchitis [15]; with digestive ailments, such as functional dyspepsia [35], irritable bowel syndrome [36, 37, 16], and esophageal symptoms [38]; with immunological disorders, such as atopy [39]. The relation of anxiety with cardiovascular diseases is less clear, some studies suggesting a link [40, 10, 12], while others contest it [41, 2, 42, 43].

Somewhat more specific in its associations is hostility, as it is mostly associated with cardiovascular diseases [44, 45] and disorders predisposing to cardiovascular disorders, such as insulin resistance [46], metabolic syndrome and atherosclerosis [47]. Few other illnesses have been proven to be linked to hostility: benign prostatic hyperplasia [48], chronic pain [49]. 

The natural question is why depression or anxiety lead to cardiovascular illnesses in some individuals, to respiratory ones in others, and to gastrointestinal ailments in yet others. Of course, some other factors (genetic, environmental, etc.) might be involved in modulating the organism's long–term response to psychoemotional disorders and in channeling its maladaptive consequences toward the cardiovascular, respiratory or gastrointestinal system. However, we are suggesting a different interpretation: features such as depression or anxiety might be too general, effacing the possibly more specific effect of certain types of depression/ anxiety or of depression/ anxiety elicited by certain causes. For example, anxiety about (means of) survival (health, money, and lodging) may be associated with disorders in some area, while social anxiety induces symptoms in another area. We have conducted a retrospective study aimed at exploring this path. In previous papers [50–55] we have described the methods we used in this retrospective study and the results we have obtained. Suffice to say that a PP has emerged for each of the main areas of pathology. We have used these PPs as the basis to conceive a triple questionnaire, the items of which have been derived from the phrases we have used to delineate these PPs.

## Meth

Our triple questionnaire consists of three questionnaires, which will be designated as Q1, Q2, and Q3 (http://drdorindragos.ro/ causesdiseases.html). To start with, we shall present only the results yielded by the first two questionnaires. Q1 and Q2 consist of 350 and 264 items respectively (there is also an on-line version, in which these 614 items are almost equally divided among three questionnaires–the links thereto may be found on the previously mentioned page). The respondents were asked to fill in the items by checking one out of five variants. The five variants are arranged in a quantitatively increasing order, such as ‘Very little/ not at all, Little, Moderately, Much, Very much’ or ‘Hardly ever/ not at all, Rarely, Occasionally, Often, Very often’.

The items are divided into four main domains, each with several subdomains. The first three domains (Preoccupation with the basal conditions of existence: health/ disease/ death, fear, money, lodging; Interaction with other people; Action, will/ volition, self–assertion) have in common the subject's tendency to self–centeredness. By contrast, the last domain (Preoccupation with the exterior) contains items exploring the state of mind of an individual whose main interest is something different from his own person. The items regarding the aforementioned four domains are randomly mixed together. Beside these psychologically oriented items, there are other items regarding various respiratory, cardiac, digestive, urinary, or gynecological symptoms and diseases the respondent may experience/ may have experienced. 

Many of the items in Q1 have their double in Q2 (i.e. for a given item in Q1 there is another one in Q2 with more or less the same meaning), in order to have the means of checking the consistency of the answers, as we consider that superficially, hastily and/ or irresponsibly answering the questionnaires might be an important factor that might alter the results.

Our aim was to discover to what degree the items of our questionnaires are representative for their subdomain and to what degree they are correlated with other items in the same subdomain, as to enable us to establish a method of testing the consistency of the answers for each respondent.

Until now, we have 3138 respondents (497 M, 2641 F), aged 33.74 [+/–] 10.99 yrs (34.96 [+/–] 12.17 yrs for males, 33.51 [+/–] 10.74 yrs for females). At this stage of our study we deemed appropriate to perform an initial analysis concerning the adequacy of our original assignment of the various items to the previously enumerated subdomains. In this first article we are presenting the results regarding the first domain (Preoccupation with the basal conditions of existence). We have estimated the correlation between each two different items pertaining to the first domain using the chi square (ChiSq) test. For the sake of clearness, we have reduced the five variants to only two: below and above the average for the respective item (for each item we have calculated the average over the whole group of respondents after assigning numerical values–1 through 5–to the five qualificatives). For each two different items a two–by–two table emerged, to which the ChiSq test with one degree of freedom was applied. The pair of items yielding 2×2 tables with at least one expected value less then 5 where dropped from analysis.

Due to the large number of calculated parameters (614 × 613 / 2 = 188191), we have considered as statistically significant only those results with an error probability (ErrProb) (evaluated by using the chi square test) of less than 10^–7^ = 0.0000001. Therefore, each of our results has a probability of at least 0.9999999 to be correct. Therefore, the probability for all of them to be correct is at least 0.9999999188191 = 0.98 (which is above the generally accepted cut–off point of 0.95). Actually, all the results we present in this paper have a ErrProb of at least 10^–20^, corresponding to a ErrProb<10^–10^ for each calculated parameter.

## Results

We are presenting the strongest correlations among items in the health subdomain (HeSD) (see Table 1), in the security subdomain (SeSD) (see Table 2), and in the stability subdomain (StSD) (see Table 3), and also between items in the HeSD and SeSD (see Table 4), between items in the HeSD and StSD (see Table 5), and between items in the SeSD and StSD (see Table 6).

**Table 1:** Correlations among items in the health subdomain (ErrProb < 10^–20^).

The correlated item in the health subdomain	ChiSq	ErrProb
‘I fear disease’ correlated to:		
I am afraid of having a serious illness	599.38	2x10^–132^
I fear death	527.7	9x10^–117^
I am worried about my health	346.74	2x10^–77^
I avoid getting into contact with sick people	157.03	5x10^–36^
I avoid touching objects of public use (handles, knobs, bannisters, handrails, etc.) lest I should catch an infection	120.68	4x10^–28^
‘I am afraid of having a serious illness’ correlated to:		
I fear death	628.37	1x10^–138^
I am worried about my health	503.99	1x10^–111^
I avoid touching objects of public use (handles, knobs, bannisters, handrails, etc.) lest I should catch an infection	106.1	7x10^–25^
‘I am worried about my health’ correlated to:		
I fear death	209.13	2x10^–47^
I undergo regular medical check–ups	138.01	7x10^–32^
I see a doctor when I experience a symptom	100.89	1x10^–^
‘I have definitively abandoned certain habits because they were harmful to my health’ correlated to:		
I have changed my diet in order to decrease the risk of becoming ill.	287.86	1x10^–64^
‘I see a doctor when I experience a symptom’ correlated to:		
I undergo regular medical check–ups	755.82	2x10^–166^
‘I undergo regular medical check–ups’ correlated to:		
I have changed my diet in order to decrease the risk of becoming ill.	116.46	4x10^–27^
‘I avoid touching objects of public use (handles, knobs, bannisters, handrails, etc.) lest I should catch an infection’ correlated to:		
I avoid getting into contact with sick people	169.55	9x10^–39^

**Table 2:** Correlations among items in the security subdomain (ErrProb < 10^–80^).

The correlated item in the security subdomain	ChiSq	ErrProb
‘I am afraid I won't have enough money to finish the building/ fitting and furnishing of my house’ correlated to:		
I fear I won't have enough money to pay my rent/ house installments	826.24	1x10^–181^
I am afraid I won't have a home of my own/ I will remain homeless	797.95	2x10^–175^
I am worried that I won't have enough money	736.3	4x10^–162^
I am afraid of running out of money	593.76	4x10^–131^
I am afraid of what the future could bring me	396.98	3x10^–88^
‘I fear I won't have enough money to pay my rent/ house installments’ correlated to:		
I am afraid of running out of money	670.15	9x10^–148^
I am worried that I won't have enough money	668.51	2x10^–147^
I am afraid I won't have a home of my own/ I will remain homeless	560.5	7x10^–124^
‘I would like to have my own house, but I don't have enough money to buy/ build one’ correlated to:		
I am afraid I won't have a home of my own/ I will remain homeless	536.79	9x10^–119^
I am preoccupied with getting a house or with furnishing and fitting of my house	459.91	5x10^–102^
‘I am worried that I won't have enough money’ correlated to:		
I am afraid I won't have a home of my own/ I will remain homeless	533.81	4x10^–118^
‘I am preoccupied with getting a house or with furnishing and fitting of my house’ correlated to:		
I am preoccupied with my house/ dwelling	710.15	2x10^–156^
I care about financial security	412.67	1x10^–91^
‘When possible, I save money for unexpected needs’ correlated to:		
I like to have savings for hard times	650.99	1x10^–143^
I carefully calculate my spendings	501.52	4x10^–111^
I have a thrifty, parsimonious nature	398.88	1x10^–88^
‘I am afraid of running out of money’ correlated to:		
I am worried that I won't have enough money	1305.07	9x10^–286^
I am worried that I won't have enough money	1305.07	9x10^–286^
I care about financial security	591.45	1x10^–130^
I am afraid I won't have a home of my own/ I will remain homeless	435.4	1x10^–96^
I am afraid of what the future could bring me	412.63	1x10^–91^
I am worried about what might happen to me	381.25	7x10^–85^
‘I am worried that I won't have enough money’ correlated to:		
I care about financial security	498.01	3x10^–110^
I am afraid of what the future could bring me	409.52	5x10^–91^
‘I am afraid of what the future might bring me’ correlated to:		
I am afraid of what the future could bring me	1450	0
I am worried about what might happen to me	683.52	1x10^–150^
There are things I am afraid of	509.09	1x10^–112^
There are events that scare me	452.11	3x10^–100^
‘I am worried about what might happen to me’ correlated to:		
I am afraid of what the future could bring me	686.01	3x10^–151^
There are things I am afraid of	513.86	9x10^–114^
There are events that scare me	475	3x10^–105^
‘There are things I am afraid of’ correlated to:		
There are events that scare me	591.72	1x10^–130^
I am afraid of darkness	414.71	3x10^–92^
I am afraid to be alone	378.28	3x10^–84^
‘I am afraid to be alone’ correlated to:		
I am afraid of darkness	431.63	7x10^–96^
‘I get frightened without knowing why’ correlated to:		
There are things I am afraid of	398.01	1x10^–88^
‘I carefully calculate my spendings’ correlated to:		
I have a thrifty, parsimonious nature	509.58	8x10^–113^
‘I waste money on things of little use’ correlated to:		
I have a thrifty, parsimonious nature	400.44	4x10^–89^
I carefully calculate my spendings	392.11	3x10^–87^

**Table 3:** Correlations among items in the stability subdomain (ErrProb < 10^–90^).

The correlated item in the stability subdomain	ChiSq	ErrProb
‘When I get home I start tidying up’ correlated to:		
I insist on having things put in order in my room/ house	545.06	1x10^–120^
I am tidy, I care for tidiness	473.75	5x10^–105^
‘Cleanliness matters to me’ correlated to:		
Dirt disgusts me	838.88	2x10^–184^
I insist on having things put in order in my room/ house.	526.42	2x10^–116^
I am tidy, I care for tidiness	518.1	1x10^–114^
When I get home I start tidying up	472.77	8x10^–105^
‘I arrange my things into categories’ correlated to:		
I am tidy, I care for tidiness	621.49	4x10^–137^
I like that everything should have a place of its own	567.04	2x10^–125^
‘I do the cleaning in my room/ house’ correlated to:		
I insist on having things put in order in my room/ house	1074.09	1x10^–235^
I am tidy, I care for tidiness	733.51	2x10^–161^
When I get home I start tidying up	591.14	1x10^–130^
‘I like out of the ordinary things’ correlated to:		
I like unusual things	935.27	2x10^–205^
‘I like unusual things.’ correlated to:		
I like to do something new, even if it's risky	528.13	7x10^–117^
I like to have savings for hard times	650.99	1x10^–143^
‘I like that everything should have a place of its own’ correlated to:		
I insist on having things put in order in my room/ house	806.02	3x10^–177^
I am tidy, I care for tidiness	763.51	5x10^–168^
‘I like events to follow a preset schedule’ correlated to:		
I like to have a definite schedule, that I know in advance	1334.02	5x10^–292^
I like to know beforehand what will happen	553.12	3x10^–122^
I like planning my activities	449.7	8x10^–100^
I like to know in advance what I have to do	447.39	3x10^–99^
‘I like when changes occur in my daily schedule’ correlated to:		
I like when changes occur in my schedule	935.88	2x10^–205^
I like unexpected things to occur	582.81	9x10^–129^
‘I like when changes occur in my schedule’ correlated to:		
I like unexpected things to occur	496.18	6x10^–110^
‘I like what is new’ correlated to:		
I like changes	617.4	3x10^–136^
‘I like to have a definite schedule, that I know in advance’ correlated		
I like to know beforehand what will happen	637.24	1x10^–140^
I like to know in advance what I have to do	490	1x10^–108^
I like planning my activities	453.49	1x10^–100^
‘I like to do something new, even if it's risky’ correlated to:		
I like what is new	468.8	6x10^–104^
I like changes	440.85	7x10^–98^
‘Before embarking on an action, I take precautions in order to leave nothing to chance’ correlated to:		
Before leaving on a trip, I study the itinerary carefully, so as to be prepared for any incidents	538.47	4x10^–119^
‘Before leaving on a trip, I study the itinerary carefully, so as to be prepared for any incidents’ correlated to:		
Before leaving on a trip, I carefully prepare the equipment	587.24	1x10^–129^
‘Dirtiness causes me repulsion’ correlated to:		
Dirt disgusts me	626.23	3x10^–138^
I insist on having things put in order in my room/ house	509.18	1x10^–112^
‘Dirt disgusts me’ correlated to:		
I insist on having things put in order in my room/ house	467.86	9x10^–104^
‘I am tidy, I care for tidiness’ correlated to:		
I insist on having things put in order in my room/ house	1263.32	1x10^–276^

**Table 4:** Correlations of health–related items with security–related items (ErrProb< 10^–30^).

The correlated item in the security subdomain	ChiSq	ErrProb
‘I fear disease’ correlated to:		
I am worried about what might happen to me	358.28	7x10^–80^
I am afraid of earthquakes	287.49	2x10^–64^
There are things I am afraid of	248.39	6x10^–56^
I am afraid of what the future might bring me	235.25	4x10^–53^
I am afraid of what the future could bring me	230.65	4x10^–52^
While on a trip in the wilderness, I am (I would be) afraid of wild animals	180.43	4x10^–41^
There are events that scare me	177.8	1x10^–40^
I am afraid to be alone	166.51	4x10^–38^
I get frightened without knowing why	149.72	2x10^–34^
I am afraid of running out of money	139.57	3x10^–32^
I am afraid of heights/ high places	137.23	1x10^–31^
‘I fear death’ correlated to:		
I am afraid of earthquakes	457.22	2x10^–101^
I am worried about what might happen to me	415.23	3x10^–92^
There are things I am afraid of	373.18	4x10^–83^
I am afraid of what the future could bring me	285.67	4x10^–64^
I am afraid of heights/ high places	249.11	4x10^–56^
I am afraid of what the future might bring me	248.69	5x10^–56^
While on a trip in the wilderness, I am (I would be) afraid of wild animals	247.96	7x10^–56^
I am afraid of darkness	246.03	2x10^–55^
I am afraid to be alone	232.25	2x10^–52^
There are events that scare me	192.95	7x10^–44^
I am afraid of wild dogs	174.02	1x10^–39^
I am afraid of snakes	167.4	3x10^–38^
I get frightened without knowing why	164.6	1x10^–37^
I am afraid of running out of money	156.9	5x10^–36^
‘I am afraid of having a serious illness’ correlated to:		
I am worried about what might happen to me	730.43	7x10^–161^
I am afraid of what the future could bring me	458.27	1x10^–101^
There are things I am afraid of	413.95	5x10^–92^
I am afraid of what the future might bring me	401.07	3x10^–89^
There are events that scare me	295.66	3x10^–66^
I am afraid of earthquakes	272.44	3x10^–61^
I am worried that I won't have enough money	268.55	2x10^–60^
I am afraid of running out of money	268.04	3x10^–60^
I am afraid I won't have enough money to finish the building/ fitting and furnishing of my house	236.94	2x10^–53^
I am afraid to be alone	232.59	2x10^–52^
I get frightened without knowing why	196.19	1x10^–44^
While on a trip in the wilderness, I am (I would be) afraid of wild animals	191.37	2x10^–43^
I am afraid of heights/ high places	188.74	6x10^–43^
I fear I won't have enough money to pay my rent/ house installments	187.57	1x10^–42^
I am afraid of darkness	182.54	1x10^–41^
I am afraid I won't have a home of my own/ I will remain homeless	182.47	1x10^–41^
I am afraid of wild dogs	148.69	3x10M^–34^
‘I am worried about my health’ correlated to:		
I am worried about what might happen to me	286.19	3x10^–64^
I am afraid of running out of money	205.21	2x10^–46^
There are things I am afraid of	191.03	2x10^–43^
I am worried that I won't have enough money	173.74	1x10^–39^
I am afraid of what the future could bring me	170.62	5x10^–39^
I care about financial security	144.15	3x10^–33^
I am afraid of what the future might bring me	143.93	4x10^–33^

**Table 5:** Correlations of health–related items with stability–related items (ErrProb< 10^–17^).

The correlated item in the security subdomain	ChiSq	ErrProb
‘I am worried about my health’ correlated to:		
I like to stick to my habits	92.17	8x10^–22^
I become attached to objects and places	86.84	1x10^–20^
It annoys me when I have to give up my usual daily activities	78.7	7x10^–19^
I care about my comfort	78	1x10^–18^
I like to know beforehand what will happen	76.22	3x10^–18^
It annoys me when people mess my room/ house	75.73	3x10^–18^
Dirt disgusts me	71.07	3x10^–17^
‘I am afraid of having a serious illness’ correlated to:		
It annoys me when I have to give up my usual daily activities	94.65	2x10^–22^
I become attached to objects and places	94.42	3x10^–22^
I feel like giving up everything and start afresh/ make a new departure	74.83	5x10^–18^
‘I fear disease’ correlated to:		
I give up to a position that I've held for a long time	78.26	9x10^–19^
I become attached to objects and places	75.13	4x10^–18^
There are things I am afraid of	373.18	4x10^–83^
I avoid going to the toilet in public places (on the train, at work, in public toilets, etc.)	69.88	6x10^–17^
‘I avoid touching objects of public use (handles, knobs, bannisters, handrails, etc.) lest I should catch an infection’ correlated to:		
I avoid going to the toilet in public places (on the train, at work, in public toilets, etc.)	249.1	4x10^–56^
Dirtiness causes me repulsion	84.52	4x10^–20^
‘I avoid getting into contact with sick people’ correlated to:		
Dirtiness causes me repulsion	86.53	1x10^–20^

**Table 6:** Correlations of health–related items with stability–related items (ErrProb< 10^–30^).

The correlated item in the security subdomain	ChiSq	ErrProb
‘I am preoccupied with my house/ dwelling’ correlated to:		
I insist on having things put in order in my room/ house	307.79	7x10^–69^
Dirt disgusts me	240.74	3x10^–54^
Dirtiness causes me repulsion	203.03	5x10^–46^
Cleanliness matters to me	201.94	8x10^–46^
I am tidy, I care for tidiness	192.86	8x10^–44^
When I get home I start tidying up	170.75	5x10^–39^
I like that everything should have a place of its own	165.15	8x10^–38^
‘I am preoccupied with getting a house or with furnishing and fitting of my house’ correlated to:		
I like to make changes in the house	179.19	7x10^–41^
I insist on having things put in order in my room/ house	178.4	1x10^–40^
Dirt disgusts me	175.53	5x10^–40^
I care about my comfort	170.86	5x10^–39^
I become attached to objects and places	144.58	3x10^–33^
Dirtiness causes me repulsion	143.93	4x10^–33^
I am tidy, I care for tidiness	141.52	1x10^–32^
I do the cleaning in my room/ house	140.02	3x10^–32^
‘I am afraid I won't have a home of my own/ I will remain homeless’ correlated to:		
I feel like giving up everything and start afresh/ make a new departure	187.15	1x10^–42^
‘I save money to buy high quality and durable items’ correlated to:		
Before embarking on an action, I take precautions in order to leave nothing to chance	190.06	3x10^–43^
‘When possible, I save money for unexpected needs’ correlated to:		
Before embarking on an action, I take precautions in order to leave nothing to chance	149.33	2x10^–34^
‘I carefully calculate my spendings’ correlated to:		
Before embarking on an action, I take precautions in order to leave nothing to chance	166.54	4x10^–38^
I like planning my activities	139.93	3x10^–32^
‘I like to have savings for hard times’ correlated to:		
Before embarking on an action, I take precautions in order to leave nothing to chance	164.36	1x10^–37^
‘I care about financial security’ correlated to:		
I care about my comfort	260.56	1x10^–58^
I like events to follow a preset schedule	150.92	1x10^–34^
‘I am worried that I won't have enough money’ correlated to:		
I feel like giving up everything and start afresh/ make a new departure	146.84	9x10^–34^
‘While searching for/ choosing a job, I take into account the wages’ correlated to:		
I feel like giving up everything and start afresh/ make a new departure	155.45	1x10^–35^
I like to know beforehand what will happen	144.44	3x10^–33^
‘I am afraid of what the future might bring me’ correlated to:		
I feel like giving up everything and start afresh/ make a new departure	172.98	2x10^–39^
‘I am afraid of what the future could bring me’ correlated to:		
I feel like giving up everything and start afresh/ make a new departure	159.9	1x10^–36^
‘There are things I am afraid of’ correlated to:		
I become attached to objects and places	151.99	6x10^–35^
‘There are events that scare me’ correlated to:		
I feel like giving up everything and start afresh/ make a new departure	142.17	9x10^–33^
‘I would like to live away from other people’ correlated to:		
I feel like giving up everything and start afresh/ make a new departure	147.55	6x10^–34^
‘I want someone to be with me’ correlated to:		
‘I want someone to be with me’ correlated to:		
I become attached to people	193.27	6x10^–44^
‘I am afraid of snakes’ correlated to:		
Dirtiness causes me repulsion	140.36	2x10^–32^

## Discussions

### Correlations among the items in the health subdomain (see Table 1)

For certain items (‘I fear disease’, ‘I am afraid of having a serious illness’, ‘I am worried about my health’, ‘I fear death’) the correlation with other items in the HeSD was not surprising. Noticeably, these items are the most general, simple and direct formulations of the health/ disease concern. As expected, there is a very high degree of correlation among these for most representative items of the HeSD, enough to confidently use these items as part of a system of evaluating the consistency of the answers received from our respondents.

Interestingly, the fear of death seems to have at least as strong correlations with the items in the HeSD as the fear of disease, although the object of the latter is a more concrete, more familiar experience. Less well correlated with the items in the HeSD are the less general items, expressing more specific attitudes that might be induced by the worry about disease.

Somehow surprising (or rather disappointing), but otherwise in line with the general trend noticed in the population, is that items expressing the eagerness to adopt active health–preserving measures (‘I have changed my diet in order to decrease the risk of becoming ill’, ‘I have definitively abandoned certain habits because they were harmful to my health’) are less well correlated with the other items in HeSD.

### The correlation of the health–related items with other subdomains

The most representative items for the HeSD seem also to correlate with the SeSD (see Table 4), almost with the same strength as with their own subdomain. That's why we were compelled to push the cut–off point for the ErrProb at 10^–30^. The best placed is ‘I am afraid of having a serious illness’, which is highly correlated with many items in the SeSD. The best correlation is with the items expressing the fear of some future unfortunate event, which is hardly surprising, as one of the most dreaded events that might happen to somebody is to get a severe illness. Quite powerful are also the correlations with items expressing general, undefined fears. It is interesting that people connect the fear of a serious disease with that of not having enough money–when confronted with ill–health; money is what we think we need (probably to pay for the treatment). Fairly strong are also the correlations with the fear of earthquake, probably because this is an uncontrollable experience definitely acknowledged as life–threatening. By contrast, there are weaker associations with fears of other definite things, which are potentially hazardous, but over which people have a much higher degree of control (animals, darkness, heights, open spaces).

The correlations of the health–related items with the StSD (see Table 5) are comparatively weak–therefore we raised the cut–off point for the probability error to 10^–17^. These associations are dominated by the items expressing the inclination to attachment and/or the avoidance of change. There are also several associations with items reflecting a repulsion for dirt–expectedly, those fearing a serious disease have a tendency to avoid uncleanliness. The strongest correlation is between two semantically similar items, both expressing the tendency to avoid direct contact with objects of public use.

### Correlations among the items in the security subdomain (see Table 2)

Although in the case of the HeSD the most general, simple and clear items correlated best with their subdomain, in the case of the SeSD the items that combine the concerns about money and dwelling are the best placed, while the simpler items (those separately reflecting the concern about financial security and about the domicile) follow. Remarkably (and counterintuitively), the most general item about dwelling (‘I am preoccupied with my house/ dwelling’) is rather poorly placed. By contrast, the better placed items are those including more specific house–related worries, such as ‘I am afraid I won't have enough money to finish the building/ fitting and furnishing of my house’. After the worries about money and house, the next best placed items are those regarding the worry about the future. And, only afterwards, come the items regarding the various other fears, firstly the fear of darkness and the fear of loneliness. People seem to fear the future more than darkness or loneliness. In the category of fears, more general items (such as ‘There are things I am afraid of’) are lower placed than more specific ones (such as the fears about money, dwelling or the future). Even lower placed are various other fears, such as of animals, of open or narrow spaces, of heights, of earthquake (ErrProb above 10^–80^ –not shown here).

Comparing these results with those obtained for the HeSD, one could wonder whether more specific disease concerns (such as fear of cancer or of heart disease) are better tools for assessing the anxiety about health than the more general items we have used in our questionnaires.

Rather amusingly, although concerns about money are at the top of the correlations list, money–earning (like ‘In searching for/ choosing a job, I take into account the wages’) or money–preserving (‘I carefully calculate my spendings’ and others) attitudes and/ or sound spending habits (for example, ‘I save money to buy high quality and durable items’) are rather at the bottom (ErrProb above 10^–40^–not shown here).

As expected, the items combining the anxiety about money and dwelling (‘I fear I won't have enough money to pay my rent/ house installments’ and ‘I am afraid I won't have enough money to finish the building/ fitting and furnishing of my house’) are best correlated with other items combining those two items, but also with items separately reflecting the financial and domiciliary concerns. Not surprising is also their correlation with the worry about the future. 

Two synonymous financial worry items (‘I am afraid of running out of money’ and ‘I am worried that I won't have enough money’) are expectedly well correlated with other money and house concerns expressing items.

No surprises as far as the house worry item ‘I am afraid I won't have a home of my own/ I will remain homeless’ is concerned: it correlates with other items regarding house and money worries.

Finally, two almost identical items reflecting the concern about the future (‘I am afraid of what the future might/ could bring me’) are indisputably well correlated, which makes the pair of them a certain candidate for the consistency evaluating system (the ErrProb is beyond the computer's calculating power– hence the zero value in the table). The worry about the future is very well correlated with a synonymous item (‘I am worried about what might happen to me’), and it is also associated with the anxiety about money and dwelling, leaving little doubt about what is the primary focus of future–related concerns.

### The correlation of the security–related items with other subdomains

Best correlated with health issues (see Table 4) are the items regarding the unexpectedness of the future and a sense of indefinite fear, but also, the concerns about money and dwelling. Some specific fears are also relevant: of earthquake and wild animals (after all, both are health hazards), but also of darkness (which may also be health–endangering if on the ill–famed streets of a metropolis).

The items in the SeSD most strongly associated with items in the StSD (see Table 6) are those regarding dwelling, money, and future. The strongest correlations are those of dwelling preoccupation with almost all the items expressing the desire for order and cleanness (a commonsensical association). Besides, people concerned about their house are also inclined to attach to objects and places (probably the house is one of the objects we are most inclined to get attached to). The financial worry items are associated with items indicating an inclination to precaution and comfort, while general fear and fear about the future are linked to attachment expressing items. Which is interesting is the link people make between snakes and dirt–it's probably engraved in the humanity’s collective unconsciousness

### Correlations among the items in the stability subdomain (see Table 3)

At the top of the correlation list there are the order–related items, followed closely and interspersed with the precaution–related ones. Less well–correlated are the cleanliness–related items, while at the bottom of the list, the items concerning the tendency to attachment (not shown here because of the low cut–off point for the ErrProb) are clustered. We should probably consider separating them as a distinct subdomain or even relocate (at least some of) them as a subdomain of a different domain (such as ‘Interaction with others’).

There are no surprises among the correlations of the most representative items for the StSD with other items in their own subdomain (see Table 3)almost all are expected, common sense correlations between almost identical or synonymous items, offering us plenty of new entries in our consistency evaluating system.

### The correlation of the stability–related items with other subdomains

There are several items associated with the HeSD (see Table 5). Expectedly, most of them are expressing the propensity to attachment, precaution, comfort, and routine. Other items in the StSD correlated with HeSD are, understandably, those pointing to a dirtiness avoiding/ abhorring attitude. There is even a very strong correlation with the public–toilet–avoiding item, explained by the synonymity with the corresponding item in the StSD. As the associations between StSD and HeSD are relatively poor, we should probably conclude that StSD is fairly distinct from the HeSD, which may raise the question about how appropriate it was to put these two subdomains in the same domain. It remains to be seen whether the analyses on larger groups of individuals confirm or otherwise this early hypothesis.

The correlation of the StSD with the SeSD is better (see Table 6): tidiness is especially linked to the concerns about house, while people prone to step on the safe side are generally concerned about money. The attachment tendency in house–worrying individuals might make us ponder about humans' natural tendency to cling to their house and property.

## Conclusions

The formulations that best express the concern about health are the most general, simple and direct ones. By contrast, the concerns about personal safety/ security are best expressed by statements combining money–and house–related worries. 

The security concerns are primarily reflected by the financial and domiciliary worries, followed by the anxiety about the future. More metaphysical fears (of darkness and of loneliness) are less prominent, and even less so are other fears, such as fear of animals, of open or narrow spaces, of heights, and of earthquake. The security concerns seem to be reflected worst by the gaining, keeping or judiciously spending money attitudes.

The inclination to stability is best expressed by the propensity to order and precaution, and less so by the desire for cleanliness. 

Regarding the items expressing the tendency to attachment, either they are poor indicators of the stability aspiration, or they reflect a different psychological trend. The tendency to attach to objects and places is more prominent among those concerned about their house.

There seems to be a substantial link between the HeSD and SeSD that primarily consists of perceiving the disease as one of the main threats to personal safety. Uncontrollable life hazards are dreaded more by health–concerned individuals than those more amenable to control. The rather loose connection between HeSD and StSD relies primarily on the attachment predisposition and uncleanliness dislike of people anxious about their health. The SeSD and StSD are associated especially due to the tidiness of dwelling concerned people and the precaution of money worried persons

Several pairs of items have emerged as safe candidates for the consistency evaluating system–in order to err on the safe side, we should probably choose the linguistically synonymous items correlated with a 10^–200^ or less ErrProb.

## References

[1] Kubzansky LD, Davidson KW (2005). The Clinical Impact of Negative Psychological States: Expanding the Spectrum of Risk for Coronary Artery Disease. Psychosom Med.

[2] Mykletun A, Bjerkeset  O, Dewey  M (2007). Anxiety, Depression, and Cause-Specific Mortality: The HUNT Study. Psychosom Med.

[3] Carney RM, Freedland KE, Steinmeyer B (2009). History of Depression and Survival After Acute Myocardial Infarction. Psychosom Med.

[4] Rutledge T (2006). Depression Is Associated With Cardiac Symptoms, Mortality Risk, and Hospitalization Among Women With Suspected Coronary Disease: The NHLBI–Sponsored WISE Study. Psychosom Med.

[5] Wulsin LR (2009). Depression and Whole Blood Serotonin in Patients With Coronary Heart Disease From the Heart and Soul Study. Psychosom Med.

[6] Surtees PG (2008). Reactive Protein, and Incident Ischemic Heart Disease in Healthy Men and Women. Psychosom Med.

[7] Suarez EC (2004). C–Reactive Protein Is Associated With Psychological Risk Factors of Cardiovascular Disease in Apparently Healthy Adults. Psychosom Med.

[8] Taylor CB (2006). Psychophysiological and Cortisol Responses to Psychological Stress in Depressed and Nondepressed Older Men and Women With Elevated Cardiovascular Disease Risk. Psychosom Med.

[9] Vaccarino V (2008). Depression, the Metabolic Syndrome and Cardiovascular Risk. Psychosom Med.

[10] Watkins LL (2006). Phobic Anxiety, Depression, and Risk of Ventricular Arrhythmias in Patients With Coronary Heart Disease. Psychosom Med.

[11] Hughes JW (2008). Depressive Symptoms Predict Heart Rate Recovery After Exercise Treadmill Testing in Patients With Coronary Artery Disease: Results From the Psychophysiological Investigations of Myocardial Ischemia Study. Psychosom Med.

[12] Bleil ME (2008). Trait Negative Affect: Toward an Integrated Model of Understanding Psychological Risk for Impairment in Cardiac Autonomic Function. Psychosom Med.

[13] Goodwin RD (2004). Respiratory Symptoms and Mental Disorders Among Youth: Results From a Prospective. Psychosom Med.

[14] Lowe B (2004). Anxiety and Depression in Patients With Pulmonary Hypertension. Psychosom Med.

[15] Wagena EJ (2004). Risk of Depression and Anxiety in Employees With Chronic Bronchitis: The Modifying Effect of Cigarette Smoking. Psychosom Med.

[16] Lackner JM (2004). Depression and Abdominal Pain in IBS Patients: The Mediating Role of Catastrophizing. Psychosom Med.

[17] Guthrie E (2004). Changes in Tolerance to Rectal Distension Correlate With Changes in Psychological State in Patients With Severe Irritable Bowel Syndrome. Psychosom Med.

[18] Mittermaier C (2004). Impact of Depressive Mood on Relapse in Patients With Inflammatory Bowel Disease: A Prospective 18–Month Follow–Up Study. Psychosom Med.

[19] Hauser W (2004). Biopsychosocial Predictors of Health–Related Quality of Life in Patients With Chronic Hepatitis C. Psychosom Med.

[20] Elwing JE (2006). Depression, Anxiety, and Nonalcoholic Steatohepatitis. Psychosom Med.

[21] Lustman PJ (2007). Depression in Diabetes: The Chicken or the Egg?. Psychosom Med.

[22] Knol MJ (2007). Depressive Symptoms in Subjects With Diagnosed and Undiagnosed Type 2 Diabetes. Psychosom Med.

[23] Weiner CL (2004). Androgens and Mood Dysfunction in Women: Comparison of Women With Polycystic Ovarian Syndrome to Healthy Controls. Psychosom Med.

[24] Bair MJ (2008). Association of Depression and Anxiety Alone and in Combination With Chronic Musculoskeletal Pain in Primary Care Patients. Psychosom Med.

[25] Thieme K (2004). Comorbid Depression and Anxiety in Fibromyalgia Syndrome: Relationship to Somatic and Psychosocial Variables. Psychosom Med.

[26] Zautra AJ (2005). Fibromyalgia: Evidence for Deficits in Positive Affect Regulation. Psychosom Med.

[27] Sherman JJ (2004). The Relationship of Somatization and Depression to Experimental Pain Response in Women With Temporomandibular Disorders. Psychosom Med.

[28] Lowe B (2004). Psychiatric Comorbidity and Work Disability in Patients With Inflammatory Rheumatic Diseases. Psychosom Med.

[29] Goodwin RD (2006). Major Depression and Allergy: Does Neuroticism Explain the Relationship?. Psychosom Med.

[30] Brzoza Z (2008). Decline in Dehydroepiandrosterone Sulfate Observed in Chronic Urticaria is Associated With Psychological Distress. Psychosom Med.

[31] Moss–Morris R (2006). To ‘Lump’ or to ‘Split’ the Functional Somatic Syndromes: Can Infectious and Emotional Risk Factors Differentiate Between the Onset of Chronic Fatigue Syndrome and Irritable Bowel Syndrome?. Psychosom Med.

[32] Katon WJ (2004). The Relationship of Asthma and Anxiety Disorders. Psychosom Med.

[33] Scott KM (2008). Childhood Adversity, Early–Onset Depressive/ Anxiety Disorders, and Adult–Onset Asthma. Psychosom Med.

[34] De Peuter S (2005). Can Subjective Asthma Symptoms Be Learned?. Psychosom Med.

[35] Van Oudenhove L (2007). Relationship Between Anxiety and Gastric Sensorimotor Function in Functional Dyspepsia. Psychosom Med.

[36] Labus JS (2007). The Central Role of Gastrointestinal–Specific Anxiety in Irritable Bowel Syndrome: Further Validation of the Visceral Sensitivity Index. Psychosom Med.

[37] Koszycki D (2005). Central Cholecystokinin Activity in Irritable Bowel Syndrome, Panic Disorder, and Healthy Controls. Psychosom Med.

[38] Naliboff BD (2004). The Effect of Life Stress on Symptoms of Heartburn. Psychosom Med.

[39] Stauder A (2003). Anxiety Symptoms in Allergic Patients: Identification and Risk Factors. Psychosom Med.

[40] Szekely A (2007). Anxiety Predicts Mortality and Morbidity After Coronary Artery and Valve Surgery–A 4–Year Follow–Up Study. Psychosom Med.

[41] Phillips AC (2009). Generalized Anxiety Disorder, Major Depressive Disorder, and Their Comorbidity as Predictors of All–Cause and Cardiovascular Mortality: The Vietnam Experience Study. Psychosom Med.

[42] Smoller JW (2006). Panic Attacks, Daily Life Ischemia, and Chest Pain in Postmenopausal Women. Psychosom Med.

[43] Rutledge T (2009). Comorbid Depression and Anxiety Symptoms as Predictors of Cardiovascular Events: Results From the NHLBI–Sponsored Women's Ischemia Syndrome Evaluation (WISE) Study. Psychosom Med.

[44] Olson MB (2005). Hostility Scores Are Associated With Increased Risk of Cardiovascular Events in Women Undergoing Coronary Angiography: A Report from the NHLBI–Sponsored WISE Study. Psychosom Med.

[45] Matthews KA (2004). Hostile behavior predicts cardiovascular mortality among men enrolled in the multiple risk factor intervention trial. Circulation.

[46] Zhang J (2006). Hostility and Urine Norepinephrine Interact to Predict Insulin Resistance: The VA Normative Aging Study. Psychosom Med.

[47] Raikkonen K (2004). Trait Anger and the Metabolic Syndrome Predict Progression of Carotid Atherosclerosis in Healthy Middle–Aged Women. Psychosom Med.

[48] Ullrich PM (2005). Stress, Hostility, and Disease Parameters of Benign Prostatic Hyperplasia. Psychosom Med.

[49] Burns JW (2006). Anger Management Style and Hostility Among Patients With Chronic Pain: Effects on Symptom–Specific Physiological Reactivity During Anger–and Sadness–Recall Interviews. Psychosom Med.

[50] Dragoş D (2008). Possible Psychological Profile of Patients with Superior Digestive Problems. Rom Med J.

[51] Dragoş D (2004). Possible Psychologic Profile of Patients with Functional Bowel Disorders. Rom Med J.

[52] Dragoş D (2009). Possible Psychologic Profile of Patients with Constipation. Rom Med J.

[53] Dragoş D (2009). A Possible Psychological Profile of Patients with Liver Disorders. Rom Med J.

[54] Dragoş D (2010). A Possible Psychological Profile of Patients with Organic Cardiac Affections. Rom J of Med Pract.

[55] Dragoş D (2010). A Possible Psychological Profile of Patients with Organic Biliary Affections. Rom J of Med Pract.

